# Multiple Comorbidities of 21 Psychological Disorders and Relationships With Psychosocial Variables: A Study of the Online Assessment and Diagnostic System Within a Web-Based Population

**DOI:** 10.2196/jmir.4143

**Published:** 2015-02-26

**Authors:** Ali M AL-Asadi, Britt Klein, Denny Meyer

**Affiliations:** ^1^School of Health SciencesSwinburne University of TechnologyHawthornAustralia; ^2^Department of Arts and EducationGrande Prairie Regional CollegeGrande Prairie, ABCanada; ^3^Centre for Biopsychosocial and eHealth Research & Innovation; DVC - Research and Innovation Portfolio; Collaborative Research Network; Faculty of HealthFederation UniversityBallaratAustralia; ^4^National Institute of Mental Health ResearchThe Australian National UniversityCanberraAustralia

**Keywords:** comorbidity, multiple comorbidities, co-occurrences, e-mental health, online, fully automated, generalized anxiety disorder, obsessive-compulsive disorder, social anxiety disorder, posttraumatic stress disorder, panic disorder, major depressive episode, insomnia, hypersomnia, dependency, alcohol, drug, suicidal ideation, social support, quality of life, sex, age

## Abstract

**Background:**

While research in the area of e-mental health has received considerable attention over the last decade, there are still many areas that have not been addressed. One such area is the comorbidity of psychological disorders in a Web-based sample using online assessment and diagnostic tools, and the relationships between comorbidities and psychosocial variables.

**Objective:**

We aimed to identify comorbidities of psychological disorders of an online sample using an online diagnostic tool. Based on diagnoses made by an automated online assessment and diagnostic system administered to a large group of online participants, multiple comorbidities (co-occurrences) of 21 psychological disorders for males and females were identified. We examined the relationships between dyadic comorbidities of anxiety and depressive disorders and the psychosocial variables sex, age, suicidal ideation, social support, and quality of life.

**Methods:**

An online complex algorithm based on the criteria of the Diagnostic and Statistical Manual of Mental Disorders, 4th edition, Text Revision, was used to assign primary and secondary diagnoses of 21 psychological disorders to 12,665 online participants. The frequency of co-occurrences of psychological disorders for males and females were calculated for all disorders. A series of hierarchical loglinear analyses were performed to examine the relationships between the dyadic comorbidities of depression and various anxiety disorders and the variables suicidal ideation, social support, quality of life, sex, and age.

**Results:**

A 21-by-21 frequency of co-occurrences of psychological disorders matrix revealed the presence of multiple significant dyadic comorbidities for males and females. Also, for those with some of the dyadic depression and the anxiety disorders, the odds for having suicidal ideation, reporting inadequate social support, and poorer quality of life increased for those with two-disorder comorbidity than for those with only one of the same two disorders.

**Conclusions:**

Comorbidities of several psychological disorders using an online assessment tool within a Web-based population were similar to those found in face-to-face clinics using traditional assessment tools. Results provided support for the transdiagnostic approaches and confirmed the positive relationship between comorbidity and suicidal ideation, the negative relationship between comorbidity and social support, and the negative relationship comorbidity and quality of life.

**Trial Registration:**

Australian and New Zealand Clinical Trials Registry ACTRN121611000704998; http://www.anzctr.org.au/trial_view.aspx?ID=336143 (Archived by WebCite at http://www.webcitation.org/618r3wvOG)

## Introduction

The comorbidity of psychological disorders is a common problem that has serious implications for the delivery of health care. The lifetime prevalence of any disorder has been reported to be 46.4%, while the lifetime prevalence of 2 and 3 disorders were found to be 27.7% and 17.3%, respectively [1]. The 12-month prevalence of any disorder was found to be 26.2%, while the 12-month prevalence of 2 and more disorders were reported to be 5.8% and 6%, respectively, and over the same period, more than 40% of those with one diagnosis met the criteria for a second diagnosis [2].

Studies on comorbidity have found strong relationships between comorbidity and higher rates of suicide [3,4], suicidal ideation [5], greater symptom severity [2,5], and poorer quality of life and social support [5]. Patients diagnosed with multiple disorders also tend to have a poorer prognosis, are less responsive to intervention, and generally exert a greater demand on the health care sector [3,4,6].

Two approaches have been used to investigate comorbidity. The co-occurrence, or frequency approach, identifies individuals with a particular diagnosis and then counts how many of them meet the diagnostic criteria of another diagnosis. The resulting comorbidity proportions therefore depend on the reference group. For example, the proportion of people diagnosed with an anxiety disorder who also meet the diagnostic criteria for an eating disorder will be different than the proportion of people diagnosed with an eating disorder who also meet the criteria for that same anxiety disorder. Moreover, because there are more people diagnosed with anxiety disorders than people diagnosed with eating disorders, it is easier to have larger samples, and hence more accuracy, when the reference group is anxiety-disordered rather than eating-disordered. The second approach identifies the psychological disorders of a group of individuals, based on discrete or dimensional scales, and then uses factor analysis to identify clusters of disorders, hence the underlying structure or dimensions of comorbidity is addressed.

**Figure 1:**
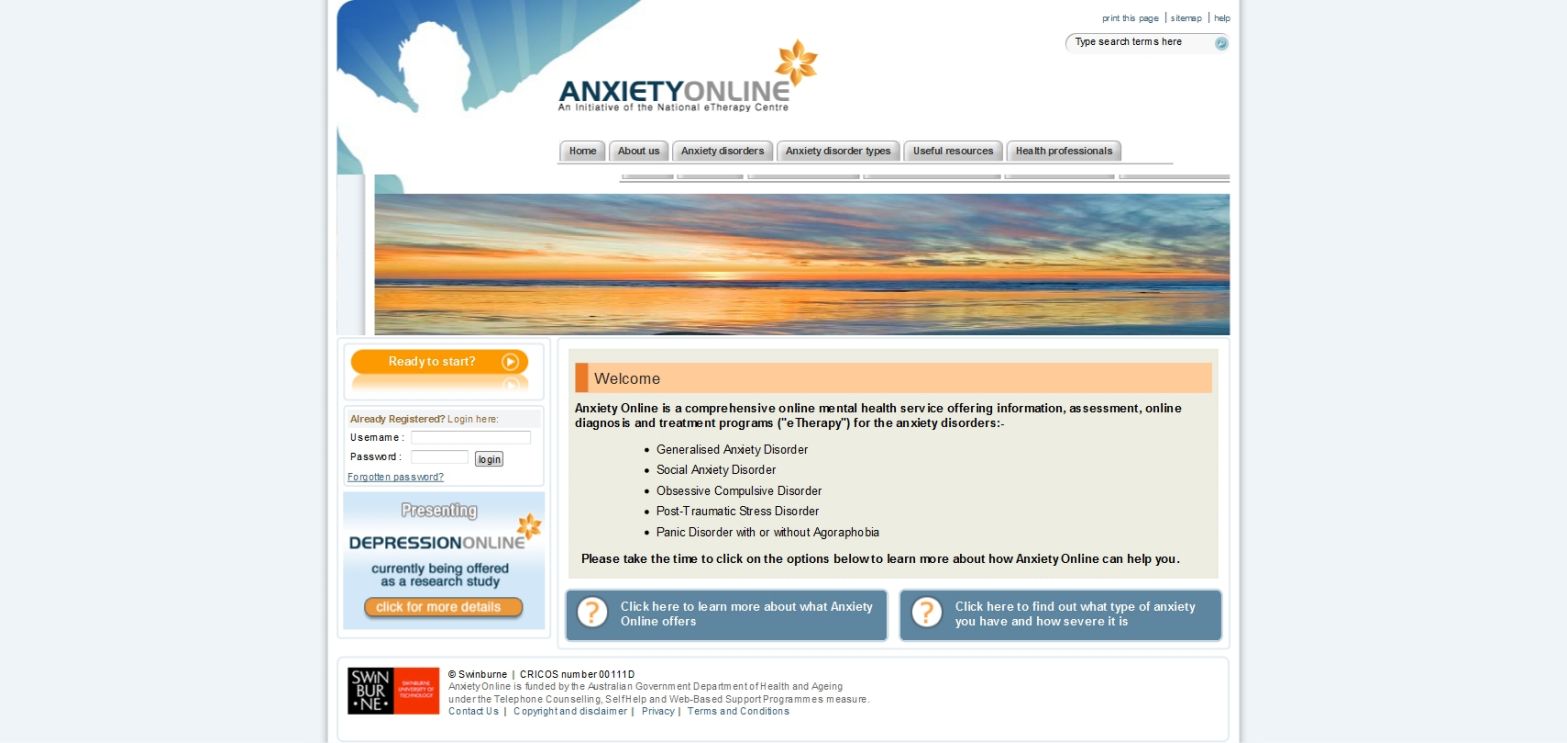
Anxiety Online homepage image.

While comorbidity, and the structure of comorbidity using in-clinic samples, has been the focus of many investigations for several decades, investigating comorbidity using an online sample is relatively new. We are not aware of any study on comorbidity using individuals who received diagnoses based on online diagnostic tools. The e-PASS data of the Mental Health Online Platform (formerly Anxiety Online) (Figure 1) [7] provide us with a unique opportunity to investigate many facets of online therapy and assessment. We have recently reported on the structure of comorbidity of 21 psychological disorders, using online diagnostic assessment, based on severity dimensional scales [5]. In this paper, we present the frequencies of co-occurrences of 21 psychological disorders using an online assessment tool within a Web-based sample and relate the identified anxiety-depression clusters to suicidal ideation, social support, and quality of life.

The comorbidities of anxiety disorders with one another are common and have long been documented, and for some anxiety diagnoses, the lack of discriminant validly was criticized [5,8-13]. The presence of anxiety disorders in clinically depressed patients is most common [14] with at least 50% of all patients diagnosed with depression meeting the diagnostic criteria for at least one anxiety disorder [15-17] and 46% of those diagnosed with major depressive disorder (MDD) showing high levels of anxiety symptoms [18]. Estimates of anxiety disorders and MDD vary based on the age of and the target population under study. For example, the comorbidity of anxiety disorders and MDD in samples of children and adolescents ranges from 15.9% to 61.9% [19,20] and 14.5% to 57% in specific populations of adult samples [21,22]. Almeida-Filho et al [23] found 74% of the depressed sample reported symptoms of anxiety disorders and 61% of those with anxiety disorders were depressed. Feva et al [24] found 50.6% of those diagnosed with MDD met the criteria for one or more anxiety disorders. Zimmerman et al [15] found 57.4% of 373 MDD outpatients meeting the criteria for at least one of the anxiety disorders. This finding was confirmed later by a meta-analysis study that concluded that 50% of individuals with MDD met the criteria for one or more anxiety disorders [25]. More specifically, Fava et al [18] found that 46% of MDD patients were significantly more likely to report symptoms associated with generalized anxiety disorder (GAD), obsessive-compulsive disorder (OCD), posttraumatic stress disorder (PTSD), agoraphobia without panic disorder (AwoPD), and panic disorder with or without agoraphobia (PD/A) than individuals without comorbid anxiety. In addition, 57% of the depressed outpatients with an anxiety disorder met the criteria for more than one anxiety disorder. In their sample, the most common comorbid anxiety disorders were social anxiety disorder (SAD) (33%), specific phobia (SP) (13.7%), PTSD (13.4%), GAD (15%), and PD/A (14.2%) [15]. Furthermore, symptoms of insomnia and hypersomnia have also been consistently present with anxiety disorders and MDD [26-30].

Although research on the comorbidity of eating disorders with other psychological disorders has produced mixed and inconsistent results, there is sufficient empirical evidence supporting the co-occurrence of eating disorders with other disorders like anxiety disorders [31,32], MDD [31,33,34], body dysmorphic disorder (BDD) [35], and substance use (drugs and alcohol) [36,37]. The estimates of these comorbidities are generally moderate to high with a wide range. For example, 55%-98% of those diagnosed with anorexia nervosa meet the diagnostic criteria for another Axis I disorder [38,39].

MDD has been found to be prevalent in individuals diagnosed with eating disorders. Estimates of the lifetime prevalence of MDD range from 50%-71% in anorexia nervosa and 50%-65% in bulimia nervosa [33,40,41]. A more recent study by Jordan et al [34] reported 63% and 51% lifetime prevalence of major depression in a 56-female anorexia nervosa sample and a 132-female bulimia nervosa sample, aged 17-40 years, respectively. On the higher end, Blinder et al [38] reported that for a female sample aged 11-68 years, 92% of the 956 patients diagnosed with anorexia nervosa and 92% of the 882 patients diagnosed with bulimia nervosa had unipolar depression disorder, while Salbach-Andrae et al [42] reported that 60% of their adolescent girls aged 12-18 years exhibited comorbid mood disorder. Conversely, among women with MDD, the lifetime prevalence rate of anorexia nervosa was estimated at 1-7% and of bulimia nervosa at 9-21% [43,44].

Anxiety disorders are also prevalent in individuals diagnosed with eating disorders, although studies on anxiety disorders and eating disorders have produced mixed results [32,45,46]. Studies that used controlled groups reported significant comorbidities between anxiety disorders and eating disorders [32,47-50]. Depending on which one of the anxiety disorders is under investigation, estimates of the lifetime prevalence of anxiety disorders in eating disorders range from as low as 0% for specific phobia in anorexia nervosa [51] to as high as 79% for OCD in anorexia nervosa [52], and from as low as 2% for agoraphobia without panic disorder in bulimia nervosa [53] to as high as 59% for SAD in bulimia nervosa [54]. A summary of life prevalence rates of MDD and various anxiety disorders in anorexia nervosa and bulimia nervosa is shown in Table 1 [33,34,38,47,51-59].

**Table 1:** Comorbidity of various anxiety disorders with anorexic nervosa and bulimia nervosa.

Anxiety disorders/MDD		Lowest, % [reference #]	Highest, % [reference #]
**Anorexia nervosa**			
	OCD	14% [51]	79% [52]
	SAD	38% [47]	55% [54]
	Agoraphobia without panic disorder	3% [54]	27% [47]
	Simple/Specific Phobia	0% [51]	45% [54]
	GAD	24% [54]	49% [47]
	PD/A	13% [47]	43% [51]
	PTSD	2% [47]	7% [47]
	MDD	46% [33] 63% [34]	74% [33] 92% [38]
**Bulimia nervosa**			
	OCD	4% [53]	43% [55]
	SAD	30% [53]	59% [54]
	Agoraphobia without panic disorder	2% [53]	35% [56]
	Simple/Specific Phobia	3% [51]	46% [57]
	GAD	2% [53]	55% [58]
	PD/A	10% [53]	53% [51]
	PTSD	5% [47]	37% [59]
	MDD	46% [33] 63% [34]	74% [33] 92% [38]

It should be noted that the time of onset for anxiety disorders, MDD, and eating disorders are not the same. Some anxiety disorders are associated with early childhood onset while others are associated with onset during adolescence. However, Pallister and Waller [46] concluded that while the relative time of onset was inconsistent, females with eating disorders exhibited higher rates of anxiety disorders compared to controls, and suggested that eating disorders and anxiety disorders might have some common underlying factors.

Another disorder that seems to be present in eating disorders, particularly in anorexia nervosa, is BDD [35,60], coupled with features of OCD engaging in ritualistic-like behaviors to reduce anxiety generated by thoughts of one’s poor self-image [35,61,62].

Finally, the substance use by individuals with psychological disorders has long been investigated [36,63,64]. The rates of substance use disorders in patients diagnosed with mood disorders and anxiety disorders were reported to be 42% and 27%, respectively [37].

In summary, the comorbidities of anxiety disorders, MDD, eating disorders, and substance use have long been established in clinical samples using traditional face-to-face assessment and diagnosis. Comorbidity estimates vary depending on the psychological disorder, age of the sample, and the specific population sample. There are no data available on the comorbidity of psychological disorders using online assessment and diagnostic tools with online samples.

The first purpose of this study is to report on the co-occurrences of 21 psychological disorders diagnosed in an online sample using an online assessment and diagnostic tool, e-PASS. While we will present the entire matrix of co-occurrences, we will focus on the comorbidities of anxiety disorders, major depressive episode (MDE), eating disorders, BDD, insomnia, hypersomnia, and alcohol use disorder. We will also report these comorbidities for males, females, separately, and together. The second purpose of this study is to examine the relationships between the identified anxiety-depression comorbidity clusters and three variables: suicidal ideation, social support, and quality of life.

## Methods

### Procedure

The Mental Health Online platform consists of four centers, one of which is the assessment center containing the e-PASS (electronic psychological assessment screening system). The e-PASS includes a variety of demographic and personal questions and the Kessler-6 and Suicidal Warnings measure, as well as the online diagnostic program. Individuals can access the Mental Health Online service from anywhere in the world provided they have an Internet connection. People can complete e-PASS if they are interested in the psychological assessment function and/or if they are interested in online treatment. Those who undertook the e-PASS were first required to register and consent to the Mental Health Online terms and conditions [7]. The procedures for collection and reporting of the Mental Health Online data were approved by the Swinburne University Human Research Ethics Committee.

### Diagnostic Assessment

Based on an individual’s response to some of the e-PASS questions, a person may be given a primary diagnosis and/or multiple secondary diagnoses. Primary or secondary diagnosis is determined by the reported presence of *Diagnostic and Statistical Manual of Mental Disorders* (4th edition, Text Revision) (DSM-IV-TR) symptoms and the average score on severity scales, each of which assesses the level of distress and interference caused by the symptoms of a particular disorder. A total of 21 clinical disorders are assessed by the e-PASS (see [5,65] for more details). The 21 psychological disorders and their abbreviations are shown in Multimedia Appendix 1.

The disorder specific severity score is the average of the scores on six questions that assess how distressed one is and how much the symptoms of a given disorder interfere in one’s life (using a scale 0=no interference/distress to 8=severe interference/distress). A person who does not endorse enough of the initial DSM-IV-TR symptom criteria questions for a particular disorder is not presented with the questions assessing their level of distress and interference of those symptoms and is assigned a severity score of zero. Those who endorse the DSM-IV symptom criteria questions for a particular disorder are presented with the six distress and interference questions allowing the calculation of an averaged severity score ranging from 0-8. An averaged distress and interference severity score of 3.5 or above is considered sufficient to warrant a clinical diagnosis. Those whose average distress and interference severity scores are less than 3.5 are considered to warrant a subclinical (or subthreshold) diagnosis.

The e-PASS diagnostic system was informed by the Anxiety Disorders Interview Schedule (ADIS) clinician rating scale (a Likert scale from 0=no symptoms, to 4=mild presence of the disorder, to 8=very severe presence of the disorder). Most “total scores” would not be a whole number because the system used six rating scales and then averaged them. Thus, 4 is the typical score by a clinician using the ADIS that indicates the “presence” of a disorder. However, considering the decimal places resulting from the e-PASS averaging of the six rating scales, those scoring 3.5 and above were deemed clinical.

The psychometric properties of the e-PASS measures were shown to have high test-retest reliability and reasonable convergent validity with the structured clinical interviews (Nguyen, unpublished PhD thesis 2013). However, the small sample size and some disagreement with the structured clinical interviews in terms of the severity levels required for a clinical diagnosis, suggest that further validation studies with large sample sizes are needed.

For the purpose of this work, we will construct a frequency matrix representing the co-occurrences of all 21 psychological disorders diagnosed by the e-PASS system. We will identify the number of participants who met the criteria for a primary disorder (reference group) and then establish the proportion of the reference group who were given a secondary diagnosis for each of the remaining 21 disorders. We will also calculate these frequencies for males and females.

### Participants

A total of 13,414 individuals completed the e-PASS phase between October 2009 and October 2012 and received at least one clinical diagnosis. The sample consisted of 3974 (29.6%) males whose ages ranged between 18-85 years old with a mean of 36.88 (SD 12.59) years, and 9440 (70.4%) females whose ages ranged between 18-86 years old with a mean of 33.66 (SD 11.57) years. A total of 749 individuals received a clinical diagnosis (severity score greater than 3.5) of only one disorder, leaving 12,665 individuals who were classified as having a clinical or subclinical diagnosis for two or more of the 21 disorders assessed by e-PASS.

### Analysis

The frequency of male and female participants who received a primary diagnosis on any particular disorder was first identified. Then, for each group, the frequencies of receiving secondary diagnoses for all 21 disorders were calculated. A series of hierarchical loglinear regression procedures were used to examine the relationships between anxiety-depression disorders and suicidal ideation, social support, and quality of life. For the significant comorbidity relationships with suicidal ideation, social support, and quality of life, demographic variables will also be explored.

## Results

### Overview

A frequency matrix of 21-by-21 disorders is shown in Multimedia Appendix 2. The number of cases of males and females receiving a primary disorder is shown in column 3 for each disorder shown column 1. Columns 4 and onward in Multimedia Appendix 2 show the number of males, females, and total and their associated percentages of those receiving secondary diagnoses. For example, of the 858 males, 1761 females, and 2649 overall who received a primary diagnosis of MDE, there were 207 (24.13%) males, 505 (28.68%) females, and 712 (26.88%) overall who received a secondary diagnosis of PD/A, respectively. Conversely, of the 478 males, 1000 females, and 1478 overall who received a primary diagnosis of PD/A, there were 289 (60.46%) males, 615 (61.50%) females, and 904 (61.16%) overall who received a secondary diagnosis of MDE, respectively.

### Comorbidities of Primary Anxiety Disorders

As shown in Multimedia Appendix 2, each anxiety disorder was comorbid with other anxiety disorders. GAD was the most comorbid anxiety disorder with other anxiety disorders ranging from 58.0% (327/564) with specific phobia to 62.31% (921/1478) with PD/A. SAD was the second most comorbid anxiety disorder with other anxiety disorders ranging from 39.5% (183/463) with OCD to 65.1% (334/513) with agoraphobia without panic disorder. The third was specific phobia with a range from 31.6% (162/513) with agoraphobia without panic disorder to 44.25% (654/1478) with PD/A. The fourth was PD/A with a range from 28.25% (378/1338) with SAD to 41.0% (231/564) with specific phobia. The fifth was PTSD with a range from 20.7% (96/463) with OCD to 31.39% (464/1478) with PD/A. The sixth was OCD with a range from 8.95% (184/2056) with GAD to 34.9% (176/504) with PTSD. The seventh was agoraphobia without panic disorder with a range from 13.8% (64/463) with OCD to 28.10% (376/1338) with SAD.

### Comorbidities of Depression, Anxiety Disorders, Insomnia/Hypersomnia, and Drug and Alcohol Abuse

We first note that there were 858 males and 1761 females who received a primary diagnosis of MDE—a 2 to 1 female to male ratio. We also note the difference in comorbidity between males and females who have been diagnosed with MDE and one of the anxiety disorders. In all cases, except for OCD, the comorbidities of MDE with PD/A, agoraphobia without panic disorder, specific phobia, PTSD, GAD, and SAD among females are greater than the comorbidity among males. In addition, about 65.50% (1735/2649) of those diagnosed with MDE suffer from insomnia, whereas about 19.29% (511/2649) suffer from hypersomnia, with females reporting symptoms of insomnia and hypersomnia in greater numbers. Also, 21.10% (559/2649) of those diagnosed with MDE report alcohol abuse with males reporting alcohol use in greater numbers.

On average, we found 58.38% of those receiving a primary diagnosis of one of the anxiety disorders also received a secondary diagnosis of MDE. Conversely, on average, we found approximately 35% of those who received a primary diagnosis of MDE also received a secondary diagnosis of one or more anxiety disorders. The lowest comorbidity was between MDE and agoraphobia without panic disorder at about 17.93% (475/2649), whereas the highest comorbidity was between MDE and GAD at about 59.61% (1579/2649). The remaining anxiety disorders in order of frequency magnitude were OCD (710/26.80%), PD/A (712/2649, 26.88%), specific phobia (803/2649, 30.31%), PTSD (883/2649, 33.33%), and SAD (1336/2649, 50.43%).

Results show the presence of the following substance dependency in participants who received a primary diagnosis of MDE: cannabis (202/2649, 7.63%), stimulant (114/2649, 4.3%), opioid (71/2649, 2.68%), sedative (252/2649, 9.51%), and alcohol (559/2649, 21.10%). Most significant was the difference between depressed males and females in alcohol dependence (25.76% (221/858) for males vs 19.19% (338/1761) for females).

The highest percentage of substance dependency present in all anxiety disorders diagnosed by e-PASS was found for males with GAD and alcohol dependence at 24.06% (141/586), for females with specific phobia and alcohol dependence at 15.10% (61/404), and for both males and females with GAD and alcohol dependence at 17.41% (358/2056).

Results indicate insomnia was present in all disorders ranging from 41.25% to 83.67% for the combined male/female samples. For males, the co-occurrence of insomnia was found highest with somatization disorder at 100% and lowest with opioid dependency and OCD at 33.33% and 33.71%, respectively. For females, the co-occurrence of insomnia was found highest with somatization disorder at 80.0% (32/40) and lowest with alcohol dependency at 42.1% (40/95).

### Comorbidities of Eating Disorders With Anxiety and Major Depressive Disorders

There were a total of 14 participants (3 males and 11 females) diagnosed with anorexia nervosa, whereas 505 participants (26 males and 479 females) diagnosed with bulimia nervosa. Results showed that 71.43% (0% males vs 90.91% females) and 71.68% (80.77% males vs 71.19% females) of those receiving a primary diagnosis of anorexia nervosa and bulimia nervosa, respectively, also received a secondary diagnosis of MDE. The co-occurrences of primary diagnosis of anorexia nervosa or bulimia nervosa with the presence of a secondary diagnosis of 1 of 7 anxiety disorders for males, females, and the total sample are extracted from Multimedia Appendix 2 and are shown in Table 2.

**Table 2:** Comorbidities of anxiety disorders, MDE in anorexia nervosa and bulimia nervosa groups.

Anxiety disorders	Anorexia nervosa, n (%)			Bulimia nervosa, n (%)		
	Male (n=3)	Female (n=11)	Total (N=14)	Male (n=26)	Female (n=479)	Total (N=505)
OCD	1 (33.3)	5 (45.5)	6 (42.9)	12 (46.2)	171 (35.7)	183 (36.2)
SAD	1 (33.3)	7 (63.6)	8 (57.1)	13 (50.0)	239 (49.9)	252 (49.9)
GAD	1 (33.3)	8 (72.7)	9 (64.3)	15 (57.7)	248 (51.8)	263 (52.1)
PD/A	0 (0.0)	3 (27.3)	3 (21.4)	5 (19.2)	126 (26.3)	131 (25.9)
PTSD	1 (33.3)	2 (18.2)	3 (21.4)	7 (26.9)	160 (33.4)	167 (33.1)
SP	0 (0.0)	4 (36.4)	4 (28.6)	9 (34.6)	127 (26.5)	136 (26.9)
AwoPD	1 (33.3)	1 (9.1)	2 (14.3)	7 (26.9)	64 (13.4)	71 (14.1)

As shown in Table 2, the comorbidity rate of anorexia nervosa with any of the 7 anxiety disorders was highest for females diagnosed with GAD at 72.7% and lowest for females diagnosed with agoraphobia without panic disorder at 9.1%. Similarly, the comorbidity rate of bulimia nervosa with any of the 7 anxiety disorders was highest for females diagnosed with GAD at 51.8% and lowest for females diagnosed with PD/A at 26.3%.

It should be noted that the rate of comorbidity depends on the reference group. For example, as shown in Multimedia Appendix 2, the rate of co-occurrence of MDE in the bulimia nervosa group was 80.8% (21/26) for males, 71.2% (341/479) for females, and 71.7% (362/505) for both. However, the rate of co-occurrence of bulimia nervosa in the MDE group was found to be 4.6% (39/858) for males, 14.82% (261/1761) for females, and 11.33% (300/2649) for both, and the rate of co-occurrence of anorexia nervosa in the MDE group was found to be 0% for males, 1% for females, and 1% for both.

### Relationships Between Comorbidities of Anxiety and Depressive Disorders and Psychosocial Variables

The data contained 6 anxiety disorders with the following primary diagnoses frequencies: GAD (n=2056), PD/A (n=1478), SAD (n=1338), specific phobia (n=564), PTSD (n=504), and OCD (n=463). The data also contained 2649 participants who received a primary diagnosis of MDE. Cross-tabulation of these 7 disorders resulted in cells with fewer than 5 participants. To maintain a cell count of 5 or greater, specific phobia, PTSD, and OCD were removed from further analyses. For the next several hierarchical loglinear analyses, MDE, GAD, PD/A, and SAD were used with each of the following variables: suicidal ideation, social support, and quality of life. In addition, age was split into young (those between 18 and 35 years old) and older (those over 35 years old). Sex of participants was also split into males and females.

### Anxiety, Depression, and Suicidal Ideation

Three anxiety disorders (PD/A, SAD, GAD) and MDE with suicidal ideation were entered into a hierarchical loglinear regression. The 5-way loglinear analyses resulted in a model with a non-significant likelihood ratio (χ^2^_10_=9.6, *P*=.476) that retained 3-way effects (χ^2^_16_=97.7, *P*<.001). Results of the backward elimination showed 2 significant triads that contained suicidal ideation: PD/A*SAD*suicidal ideation and PD/A*MDE*suicidal ideation. Two new hierarchical loglinear models were constructed. The first model was based on PD/A, SAD, suicidal ideation, age, and sex that resulted in a non-significant likelihood ratio (χ^2^_10_=12.7, *P*=.240) that retained 3-way effects (χ^2^_16_=81.5, *P*<.001). The second model was based on PD/A, MDE, suicidal ideation, age, and sex which resulted in a non-significant likelihood ratio (χ^2^_10_=6.5, *P*=.772) that retained 3-way effects (χ^2^_16_=34.8, *P*=.004). Consequently, a new model with PD/A, SAD, MDE, suicidal ideation, age, and sex with only 3-way effects resulted in a non-significant likelihood ratio (χ^2^_32_=22.4, *P*=.90). Backward elimination resulted in 2 significant triads that included suicidal ideation, MDE-PD/A comorbidity dyad and PD/A-SAD comorbidity dyad, as shown in Table 3. A 2x2 cross-tabulation for those who reported no suicidal ideation and for those who reported suicidal ideation was performed separately for the MDE-PD/A comorbidity dyad and for the PD/A-SAD comorbidity dyad.

**Table 3:** Values and significance of chi square test for 3-way interactional terms for MDE, PD/A, SAD, sex, and age with suicidal ideation.

3-way interaction	χ^2^(df=1)	*P*
MDE*PD/A*SAD	13.7	.000
MDE*PD/A*suicidal ideation	7.6	.006
MDE*SAD*Sex	8.3	.004
PD/A*SAD*Suicidal ideation	14.3	.000
PD*SAD*Age	22.7	.000
Suicidal Ideation*Sex*Age	6.0	.014

### MDE-PD/A Comorbidity Dyad

**Figure 2:**
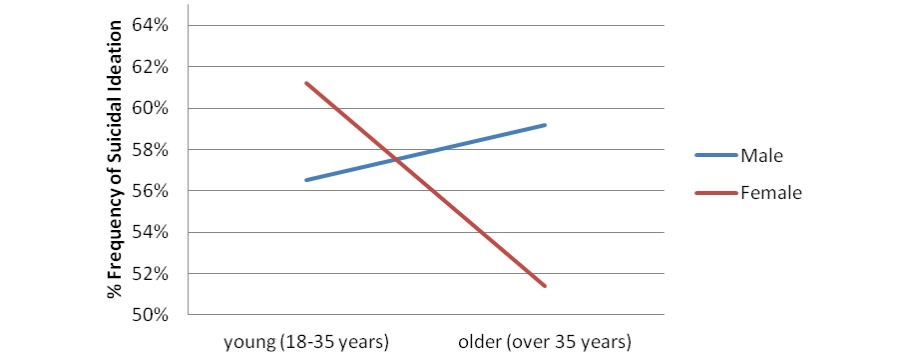
Sex by age for those who endorsed suicidal ideation (% frequency) and endorsed PD/A and MDE.

For the non-suicidal ideation group, there was a significant association between MDE and whether or not PD/A was endorsed (χ^2^_1_=11.1, *P*=.001). The odds for the non-suicidal ideation group endorsing both MDE and PD/A were 1.18 times higher than if they had endorsed MDE only. For the suicidal ideation group, there was a significant association between MDE and whether or not PD/A was endorsed (χ^2^_1_=21.0, *P*<.001). This was based on the finding that the odds of the suicidal ideation group endorsing both MDE and PD/A were 1.84 times higher than if they had endorsed MDE only. The frequencies of all combinations of PD/A, MDE, sex, and age of those who reported suicidal ideation are shown in Table 4, and a graph illustrating the effect of sex and age on suicidal ideation when there is PD/A and MDE comorbidity is shown in Figure 2.

**Table 4:** Frequencies (%) of those endorsing suicidal ideation by PD/A*MDE*Sex*Age.

PD/A	MDE	Sex	Age	Suicidal ideation, n/N (%)
Yes	Yes	M	Y (≤35)	309/547 (56.5)
Yes	Yes	M	O (>35)	266/449 (59.2)
Yes	Yes	F	Y	1075/1757 (61.18)
Yes	Yes	F	O	416/809 (51.4)
Yes	No	M	Y	15/183 (8.2)
Yes	No	M	O	12/170 (7.1)
Yes	No	F	Y	40/550 (7.3)
Yes	No	F	O	18/314 (5.7)
No	Yes	M	Y	455/908 (50.1)
No	Yes	M	O	416/883 (47.1)
No	Yes	F	Y	1194/2419 (49.36)
No	Yes	F	O	676/1547 (43.70)

### PD/A-SAD Comorbidity Dyad

**Figure 3:**
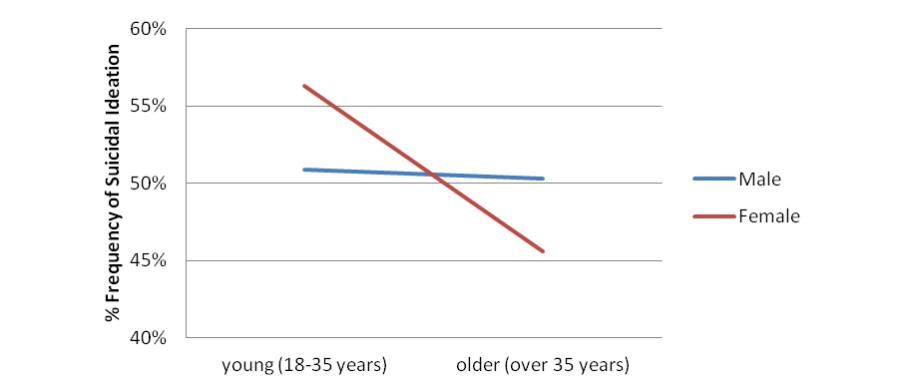
Sex by age for those who endorsed suicidal ideation (% frequency) and endorsed PD/A and SAD.

For the non-suicidal ideation group, there was a significant association between PD/A and whether or not SAD was endorsed (χ^2^_1_=34.6, *P*<.001). The odds of the non-suicidal ideation group endorsing both PD/A and SAD were 1.32 times higher than if they had endorsed PD/A only. For the suicidal ideation group, there was a significant association between PD/A and whether or not SAD was endorsed by this group (χ^2^_1_=140.4, *P*<.001). The odds of the suicidal ideation group endorsing both PD/A and SAD were 2.07 times higher than if they had endorsed PD/A only. The frequencies of all combinations of PD/A, SAD, sex, and age of those who reported suicidal ideation are shown in Table 5, and a graph of the effect of age and sex on suicidal ideation in the presence of a PD/A and SAD comorbidity is shown in Figure 3.

**Table 5:** Frequencies (%) of those endorsing suicidal ideation by PD/A*SAD*Sex*Age.

PD/A	SAD	Sex	Age	Suicidal ideation, n/N (%)
yes	yes	M	Y (≤35)	244/479 (50.9)
yes	yes	M	O (>35)	197/392 (50.28)
yes	yes	F	Y	842/1496 (56.3)
yes	yes	F	O	314/689 (45.6)
yes	no	M	Y	80/251 (31.9)
yes	no	M	O	81/227 (35.7)
yes	no	F	Y	273/811 (33.7)
yes	no	F	O	120/434 (27.6)
no	yes	M	Y	303/776 (39)
no	yes	M	O	247/633 (39)
no	yes	F	Y	804/2021 (39.78)
no	yes	F	O	361/1049 (34.41)

### Anxiety, Depression, and Social Support

Three anxiety disorders (PD/A, SAD, GAD) and MDE with social support were entered into a hierarchical loglinear regression. The 5-way loglinear resulted in a model, with a non-significant likelihood ratio (χ^2^_10_=7.1, *P*=.72), which retained 3-way effects (χ^2^_16_=86.5, *P*<.001). Results of the backward elimination showed 1 significant triad that contained social support, PD/A*GAD*social support (χ^2^_1_=4.3, *P*=.038). The remaining 4 significant triads were the 3-way interactions of MDE*GAD*PD/A, MDE*GAD*SAD, GAD*PD/A*SAD, and MDE*PD/A*SAD that did not include social support. A hierarchical loglinear model was constructed using PD/A, GAD, and social support with sex and age. The resulting model had a non-significant likelihood ratio (χ^2^_10_=2.2, *P*=.994) that retained 3-way effects (χ^2^_16_=40.4, *P*=.001). A new model with only 3-way effects resulted in a non-significant likelihood ratio (χ^2^_16_=17.7, *P*=.35). Backward elimination resulted in one significant triad that included social support and GAD-PD/A comorbidity dyad, as shown in Table 6.

**Table 6:** Values and significance of chi square test for 3-way interactional terms for GAD, PD/A, sex, and age with social support.

3-way interaction	χ^2^(df=1)	*P*
GAD*PD/A*Social Support	9.6	.002
GAD*Sex*Age	19.6	.000
PD*Age	42.5	.000
Social Support*Sex	14.9	.000

A 2x2 cross-tabulation for those who reported inadequate social support and for those who reported adequate social support was performed separately for the GAD-PD/A comorbidity dyad. For those who reported inadequate social support, there was a significant association between GAD and whether or not PD/A was endorsed (χ^2^_1_=119.12, *P*<.001). This was based on the finding that the odds of inadequate social support group endorsing both GAD and PD/A were 1.84 times higher than if they had endorsed GAD only. For those who reported adequate social support, there was a significant association between GAD and whether or not PD/A was endorsed (χ^2^_1_=40.31, *P*<.001). The odds of the adequate social support group endorsing both GAD and PD/A were 1.42 times higher than if they had endorsed GAD only. Table 7 shows the frequencies of all combinations of PD/A, GAD, sex, and age of those who endorsed adequate social support.

**Table 7:** Frequencies (%) of those endorsing social support by PD/A*GAD*Sex*Age.

PD/A	GAD	Sex	Age	Social support, n/N (%)
Yes	Yes	M	Y (≤35)	189/507 (37.3)
Yes	Yes	M	O (>35)	166/435 (38.2)
Yes	Yes	F	Y	671/1744 (38.47)
Yes	Yes	F	O	304/793 (38.3)
Yes	No	M	Y	116/223 (52.0)
Yes	No	M	O	97/184 (52.7)
Yes	No	F	Y	294/563 (52.2)
Yes	No	F	O	193/330 (58.5)
No	Yes	M	Y	301/781 (38.5)
No	Yes	M	O	306/787 (38.9)
No	Yes	F	Y	1032/2362 (43.69)
No	Yes	F	O	639/1421 (44.97)

### Anxiety, Depression, and Quality of Life

Three anxiety disorders (PD/A, SAD, GAD) and MDE with quality of life were entered into a hierarchical loglinear regression. The 5-way loglinear resulted in a model, with a non-significant likelihood ratio (χ^2^_10_=11.1, *P*=.35) that retained 3-way effects (χ^2^_16_=92.6, *P*<.001). Results of the backward elimination showed 1 significant triad that contained quality of life, PD/A*SAD*quality of life (χ^2^_1_=4.6, *P*=.031). The remaining 3 significant triads were the 3-way interactions of MDE*PD/A*SAD, MDE*GAD*SAD, and MDE*GAD*PD/A that did not include quality of life. A hierarchical loglinear model was constructed using PD/A, SAD, and quality of life with sex and age. The resulting model had a non-significant likelihood ratio (χ^2^_10_=7.7, *P*=.66) that retained 3-way effects (χ^2^_16_=56.0, *P*<.001). A new model with only 3-way effects resulted in a non-significant likelihood ratio (χ^2^_13_=14.1, *P*=.37). Backward elimination resulted in one significant triad that included quality of life and PD/A-SAD comorbidity dyad. The rest of the 5 significant triads did not include any comorbidity dyad with the quality of life term, as shown in Table 8.

**Table 8:** Values and significance of chi square test for 3-way interactional terms for SAD, PD/A, sex, and age with quality of life.

3-way interaction	χ^2^(df=1)	*P*
PD*Quality of Life*SAD	16.4	.000
PD*Quality of Life*Sex	4.1	.042
PD*SAD*Age	18.0	.000
PD*Sex*Age	4.2	.040
SAD*Sex	5.4	.020

A 2x2 cross-tabulation for those who reported poor quality of life and for those who reported good quality of life was performed separately for PD/A-SAD comorbidity dyad. For those who reported poor quality of life, there was a significant association between PD/A and whether or not SAD was endorsed (χ^2^_1_=130.1, *P*<.001). This was based on the finding that the odds of poor quality of life group endorsing both PD/A and SAD were 1.87 times higher than if they had endorsed PD/A only. For those who reported good quality of life, there was a significant association between PD/A and whether or not SAD was endorsed by this group (χ^2^_1_=34.3, *P*<.001). The odds of the good quality of life group endorsing both PD/A and SAD were 1.35 times higher than if they had endorsed PD/A only. The frequencies of all combinations of PD/A, SAD, sex, and age of those who endorsed good quality of life are shown in Table 9.

**Table 9:** Frequencies (%) of those endorsing quality of life by PD/A, SAD, sex, and age.

PD/A	SAD	Sex	Age	Quality of life, n/N (%)
Yes	Yes	M	Y (≤35)	188/479 (39.2)
Yes	Yes	M	O (>35)	145/392 (37.0)
Yes	Yes	F	Y	567/1496 (37.90)
Yes	Yes	F	O	270/689 (39.2)
Yes	No	M	Y	147/251 (58.6)
Yes	No	M	O	113/227 (54.4)
Yes	No	F	Y	503/811 (62.0)
Yes	No	F	O	253/434 (58.3)
No	Yes	M	Y	339/776 (43.7)
No	Yes	M	O	386/663 (45.2)
No	Yes	F	Y	1011/2021 (50.02)
No	Yes	F	O	519/1049 (49.48)

## Discussion

### Principal Findings

A frequency matrix of the co-occurrence of 21 psychological disorders based on primary and secondary diagnoses of 12,665 individuals who were assessed using the e-PASS online diagnostic system was constructed. To the best of our knowledge, such a matrix for this many psychological disorders has not been presented before for traditional in-clinic diagnosis or for any online diagnostic tools. As such, comparisons with existing literature should be viewed with caution. We present this matrix to serve as a preliminary and potentially useful reference for future works in the area of online assessment and diagnosis.

Given the high number of disorders in the matrix and limited discussion space, we will focus on a few disorders that are of most interest and/or have been studied before in in-clinic samples.

### Depression, Anxiety Disorders, Insomnia/Hypersomnia, and Drug and Alcohol Abuse

One area that has been studied extensively is the comorbidity of MDE and the various anxiety disorders, and alcohol abuse. We found the number of females receiving a primary diagnosis of MDE was twice the number of males receiving a primary diagnosis of MDE. This ratio of about 2:1 females to males is consistent with in-clinic samples and face-to-face diagnostic tools. We also found the comorbidities of MDE with all anxiety disorders, except for OCD, among females to be greater than the same comorbidities among males. In addition, the majority (2 in 3) of those diagnosed with MDE reported insomnia, whereas 1 in 5 reported hypersomnia, with greater numbers of females than males reporting symptoms of insomnia and hypersomnia. Moreover, 1 in 5 of those diagnosed with MDE reported alcohol abuse with greater numbers of males than females reporting alcohol abuse.

On average, we found approximately 1 in 3 of those who received a primary diagnosis of MDE also received a secondary diagnosis of one or more anxiety disorders. The lowest comorbidity was found between MDE and agoraphobia without panic disorder whereas the highest comorbidity was between MDE and GAD. These results are consisted with findings based on in-clinic samples [15,21,24,25,66]. Our findings are also consistent with Fava et al [24] who found that 46% of MDD patients were significantly more likely to report symptoms associated with GAD, OCD, PTSD, agoraphobia without panic disorder, and PD/A than individuals without comorbid anxiety. On the higher end, Almeida-Filho et al [23] found 74% of a depressed Brazilian sample reported symptoms of anxiety disorders, which is much higher than our results of 35%. This discrepancy is possibly due to the fact that they used reported symptoms of anxiety disorders, whereas this study used adherence to the DSM-IV-TR diagnostic criteria. On the other hand, our result that almost 2 in 3 of those receiving a primary diagnosis of one of the anxiety disorders was also receiving a secondary diagnosis of MDE is consistent with Almeida-Filho et al’s [23] results that 61% of those with anxiety disorders were depressed.

We also found high rates of comorbidities among anxiety disorders with GAD being the most comorbid anxiety disorder with other anxiety disorders followed by SAD, specific phobia, PD/A, PTSD, OCD, and agoraphobia without panic disorder. These results are consistent with previous research findings that found anxiety disorders to have high comorbidities with each other and that questioned the discriminant validity of some anxiety diagnoses such as GAD [5,8-13]. This consistency may suggest that there are no differences between online and in-clinic assessment systems and online and in-clinic populations.

The comorbidities between MDE and various substance dependency (ranging between 3% to 21%) found in this study are much lower than the 42% of mood disordered patients who had substance use disorders as reported by McGovern et al [37]. Again, this discrepancy may be due to this study’s strict adherence to the diagnostic criteria.

The highest comorbidity of substance dependency present in all anxiety disorders was found for males who received a primary diagnosis of GAD and a secondary diagnosis of alcohol dependence at 24.06%, and for females who received a primary diagnosis of specific phobia and a secondary diagnosis of alcohol dependence at 15.10%. McGovern et al [37], without examining males and females separately, found substance use disorders present in 27% of patients diagnosed with anxiety disorders, which is, again, slightly greater than results of this study of about 17% for the combined male and female samples. Almeida-Filho et al [23] found 20% of cases of alcoholism co-occurring with anxiety disorders and MDD diagnoses.

This study also found insomnia to be present in all disorders ranging from 41.25% to 83.67% for the combined male/female samples across all disorders. This association between insomnia and psychological disorders is consistent with the literature. For example, the presence of sleep problems has been consistently found in patients with anxiety and mood disorders [5,26-30].

### Eating Disorders, Anxiety Disorders, and Major Depression

The interpretation of the comorbidities of anorexia nervosa with other disorders should be viewed with caution because of the small number of participants who were diagnosed with anorexia nervosa. Overall, our results are consistent with the previously found rates of anorexia nervosa and bulimia nervosa comorbidities with MDE and anxiety disorders. Specifically, we found 71.43% and 71.68% of those receiving a primary diagnosis of anorexia nervosa and bulimia nervosa, respectively, also received a secondary diagnosis of MDE. The comorbidity of anorexia nervosa and MDE found in this study is within the range of 50%-71% reported by previous studies [33,34,40-42]. However, the result for the comorbidity of bulimia nervosa and MDE found in this study is slightly greater than the range of 50%-65% reported by previous investigations [33,34,40,41].

The rates of co-occurrence of bulimia nervosa and anorexia nervosa in the MDE group found in this study are consistent with the estimated lifetime prevalence of anorexia nervosa in MDD (1%-7%) and of bulimia nervosa in MDD (9%-21%) reported by Carter et al [43] and Fava et al [44]. It is expected that a larger proportion of bulimia nervosa individuals experience symptoms of depression while a much smaller percentage of depressed individuals experience symptoms of bulimia nervosa.

While the choice of the reference group is very important in establishing comorbidity rates, it varies in importance. For example, while defining the reference group in the case of MDE or anxiety disorders and bulimia nervosa or anorexia nervosa is important, it is less so when defining the reference group for MDE and GAD. As shown in Multimedia Appendix 2, the co-occurrence of MDE in the GAD group is 66.83% whereas the co-occurrence of GAD in the MDE group is 59.61% for combined male and female sample.

For the most part, results of this study are consistent with previous findings. However, results do not fall within the range found in previous studies on three occasions. The comorbidity rate for anorexia nervosa with GAD (64%) found by this study is outside the range of 24%-49% reported by Godart et al [47,54]. Similarly the comorbidity rate for anorexia nervosa with PTSD (21%) is outside the range of 2%-7% given by Godart et al [47]. In both cases, the results of this study are based on very few participants with anorexia nervosa and therefore should be interpreted with caution. Finally, the comorbidity rate for bulimia nervosa with MDE (72%) is slightly outside the range of 61%-65% reported by Jordan et al [34] and Casper [33].

This study also examined the interactional relationships between GAD, SAD, PD/A, MDE, sex, and age and three variables: suicidal ideation, social support, and quality of life using a series of hierarchical loglinear analyses. In each case, 3-way interaction effects were found. For suicidal ideation, the odds of endorsing having suicidal ideation was greater for those diagnosed with depression and PD/A than depression only, and for those diagnosed with PD/A and SAD than PD/A only. These results suggest that comorbidity, even for two disorders, increases the risk of having suicidal thoughts, as indicated by previous research [3-5]. We also found a significant interactional effect for sex by age. The frequency of younger females (18-35 years old) diagnosed with MDE and PD/A or PD/A and SAD endorsing suicidal ideation was about 10% greater than older females (over 35 years old) and about 5% greater than their counterpart younger or older males. These results suggest that younger females who have these comorbidity dyads are at greater risk of having suicidal ideation.

The results of this study found the GAD and PD/A dyad to be the only one to have a significant relationship with social support. The odds for reporting having inadequate social support was greater for those diagnosed with GAD and PD/A than GAD only. There are not many studies that examined the relationship between comorbidity and social support, but one recent study reported a negative relationship between comorbidity and social support [5]. We should note here that this study found no significant interactional effect between GAD and PD/A comorbidity dyad and sex, age, and social support. These results suggest that sex and age have little effect on the relationship between this dyadic comorbidity and social support.

Finally, the results suggested that the PD/A and SAD dyad was the only dyad to have a significant relationship with quality of life. The odds for reporting having a poor quality of life was greater for those diagnosed with PD/A and SAD than PD/A only. Again, only one study reported a negative relationship between comorbidity and quality of life [5]. Also, as was the case with social support, we found no significant interactional effect between the PD/A-SAD comorbidity dyad and sex, age, and quality of life. These results suggest that sex and age have no effect on the relationship between this dyadic comorbidity and quality of life.

### Transdiagnostic Approaches

There is growing support for using transdiagnostic approaches for the assessment and treatment of psychological disorders. AL-Asadi et al [5] using dimensional scales found overlapping dimensions underlying the various psychological disorders. Moses and Barlow [67] and Barlow et al [68] concluded that at a minimum, a diagnostic specific approach and transdiagnostic approaches to treatments are equally effective. AL-Asadi et al [69] found significant reduction in the severity of symptoms of depression as a result of participants receiving anxiety-specific treatment and supported the efficacy of online therapy to provide transdiagnostic treatment. McEvoy et al’s [70] review of the literature concluded that transdiagnostic treatments were associated with improvements in comorbidity disorders and with high client satisfaction, therapeutic alliance, group cohesion, and positive treatment expectations. McManus et al [71] pointed out the potential of transdiagnostic approaches in addressing multiple comorbid anxiety disorders. Wade et al [72] found support for using transdiagnostic approaches to understanding eating disorders. Results of this study provide further support for the use of transdiagnostic approaches to the assessment and treatment of psychological disorders.

### Limitations

One of the major limitations of this study is the lack of a control group. The online system does not require the inclusion of a control group and consequently any conclusion must be taken as preliminary. Another limitation is the lack of research on the sensitivity and the psychometric properties of the e-PASS system. There is only one unpublished study that found the e-PASS system to have high test-retest reliability and adequate convergent validity (Nguyen, unpublished PhD thesis, 2013). Unfortunately, even this one study has used a small sample size and found disagreement between e-PASS and structured clinical interviews when it came to the level of severity required for a clinical diagnosis. More validation studies with larger samples and using the newly released DSM-5 criteria are required before definitive conclusions can be made. The last limitation is inherent to all self-report instruments such as e-PASS. The exclusive reliance of e-PASS on automated online self-report measures brings into question the extent to which diagnosing individuals is reliable. Concerns have been raised regarding the reliability of online diagnostic tools [73].

### Conclusions

In summary, overlap between psychological disorders for our online sample using the online assessment tool, e-PASS, was confirmed and was found to be similar to in-clinic samples using face-to-face assessment tools. Overall, there did not appear to be much difference in the rates of comorbidities of psychological disorders between in-clinic samples using face-to-face assessment and diagnostic tools and our online sample using the online assessment and diagnostic tool, e-PASS. The results of this study showed that the comorbidity rates for the online sample using e-PASS commonly fell within the range found for in-clinic samples using in-clinic assessment tools. The observation that e-PASS and face-to-face assessment tools generally yielded the same result may provide further evidence to the validity and the utility of the Anxiety Online Platform and the e-PASS assessment tool. Findings of this study supported the use of transdiagnostic approaches in the assessment and treatment of psychological disorders. Moreover, dyadic disorder comorbidities of some anxiety disorders and MDE were found to increase the odds for having suicidal ideation, inadequate social support, and poorer quality of life than a diagnosis of only one of the two making up the dyadic disorder.

## Multimedia Appendix 1

Abbreviations for the 21 psychological disorders diagnosed by e-PASS.

## Multimedia Appendix 2

Table - Frequency of co-occurrences of 21 psychological disorders for males, females, and both.

## Abbreviations

ADIS: Anxiety Disorders Interview Schedule
AwoPD: agoraphobia without panic disorder
BDD: body dysmorphic disorder
DSM-5: Diagnostic and Statistical Manual of Mental Disorders (5th edition)
DSM-IV-TR: Diagnostic and Statistical Manual of Mental Disorders (4th edition, Text Revision)
e-PASS: electronic psychological assessment screening system
GAD: generalized anxiety disorder
MDD: major depressive disorder
MDE: major depressive episode
OCD: obsessive compulsive disorder
PD/A: panic disorder with or without agoraphobia
PTSD: posttraumatic stress disorder
SAD: social anxiety disorder
SP: specific phobia
