# Supplementary material for: Multiple Comorbidities of 21 Psychological Disorders and Relationships With Psychosocial Variables: A Study of the Online Assessment and Diagnostic System Within a Web-Based Population
Source: J Med Internet Res. 2015 Feb 26;17(3):e55. doi: 10.2196/jmir.4143 (PMC4392551; doi:10.2196/jmir.4143)
Supplement: Supplementary file 1 [file jmir_v17i3e55_app1.pdf]

Appendix "1"

Abbreviations for the 21 Psychological Disorders Diagnosed by e-PASS

PD/A	Panic Disorder with or without Agoraphobia		MDE	Major Depressive Episode		SD_SED	Substance Dependence - Sedatives
AwoPD	Agoraphobia w/o a history of Panic Disorder		AN	Anorexia Nervosa		ALCD	Alcohol Dependence
SAD	Social Anxiety Disorder		BN	Bulimia Nervosa		SMD	Somatization Disorder
SP	Specific Phobia		BED	Binge Eating Disorder		BDD	Body Dysmorphic Disorder
GAD	Generalized Anxiety Disorder		SD_CAN	Substance Dependence - Cannabis		PG	Problem / Pathological Gambling
OCD	Obsessive-Compulsive Disorder		SD_ST	Substance Dependence - Stimulants		PI	Insomnia
PTSD	Post Traumatic Stress Disorder		SD_OP	Substance Dependence - Opioids		PH	Hypersomnia
